# Properties, X-ray data and 2D WAXD fitting procedures of melt-spun poly(ɛ-caprolactone)

**DOI:** 10.1016/j.dib.2020.106223

**Published:** 2020-08-24

**Authors:** F. Selli, A. Gooneie, U.H. Erdoğan, R. Hufenus, E. Perret

**Affiliations:** a Laboratory for Advanced Fibers, Empa, Swiss Federal Laboratories for Materials Science and Technology, Lerchenfeldstrasse 5, 9014 St. Gallen, Switzerland; b Department of Textile Engineering, Dokuz Eylul University, Buca-Izmir, Turkey; c Center for X-ray Analytics, Empa, Swiss Federal Laboratories for Materials Science and Technology, Überlandstrasse 129, 8600 Dübendorf, Switzerland

**Keywords:** Poly(ԑ-caprolactone), Melt-spinning, Mesophase, Biodegradable fiber, SAXS, WAXD simulation

## Abstract

Rheological and thermal properties of the poly(ɛ-caprolactone) (PCL) polymer are presented in Section 1.1. Section 1.2 summarizes results of melt-spun PCL filaments. Specifically, we show the necking point stabilization during high-speed online drawing in Section 1.2.1, filament morphology in Section 1.2.2, wide-angle X-ray diffraction (WAXD) fitting results in Section 1.2.3, WAXD patterns of aged fibers in Section 1.2.4, crystallinity analysis in Section 1.2.5 and small-angle X-ray scattering (SAXS) analysis results in Section 1.2.6. Details about the materials, experimental and analytical methods are given in Section 2. Of particular interest may be the simulation and fitting procedures of 2D WAXD patterns, which are summarized in Section 2.7.2. For more information see the publication by Selli et al. 'Mesophase in melt-spun poly(ɛ-caprolactone) filaments: structure–mechanical property relationship' [1].

## Specifications Table

**Table:** 

Subject	Materials Science: Polymers and Plastics
Specific subject area	Biodegradable melt-spun monofilaments.
Type of data	Table Image Figure Equations
How data were acquired	Instruments: Rheometer Physica MCR 301 (Anton Paar) DSC 214 Polyma, Netzsch, Selb, Germany TG 209 F1, Netzsch, Selb, Germany FE-SEM S-4800 (Hitachi High-Technologies Europe, Krefeld, Germany) Bruker Nanostar U diffractometer Software: DIFFRAC.EVA (version 4.2., Bruker AXS, Karlsruhe, Germany) Python codes NETZSCH Proteus software Rheoplus/32 V3.40
Data format	Raw Analyzed
Parameters for data collection	Rheological and thermal properties were measured from the PCL polymer. Morphological properties of melt-spun PCL filaments were analysed with a scanning electron microscope. Wide-angle X-ray diffraction (WAXD) and small-angle X-ray scattering (SAXS) patterns were taken from online and offline drawn melt-spun PCL filaments with a Bruker Nanostar U diffractometer.
Description of data collection	Rheological properties of the PCL polymer were analyzed with a rheometer using a plate-plate geometry. Thermal properties of the PCL polymer were analyzed with DSC and TGA. SEM pictures were taken from the surfaces of the melt-spun PCL filaments. WAXD and SAXS patterns of online and offline drawn melt-spun PCL filaments were recorded on a Bruker Nanostar U diffractometer (Bruker AXS, Germany) with Cu-Kα radiation (λ = 1.5419 Å) and a VÅNTEC-2000 MikroGap area detection system. A beam defining pinhole of 300 µm was used. The WAXD and SAXS measurements were performed in two separate experiments with distances of 19.1 cm and 110.1 cm, respectively, between sample and active detector area. WAXD patterns were subsequently fit with python codes. Transversal and meridional scans were extracted from SAXS patterns and fitted in order to extract structural information.
Data source location	Empa, St. Gallen, Switzerland
Data accessibility	Mendeley Data DOI: 10.17632/rb64282 × 5p.2 http://dx.doi.org/10.17632/rb64282X5p.2
Related research article	F. Selli, U.H. Erdogan, R. Hufenus, E. Perret Mesophase in melt-spun poly(ɛ-caprolactone) filaments: structure–mechanical property relationship Polymer DOI: (under review)

## Value of the Data

• The understanding of the structure of melt-spun PCL fibers is of great value for biomedical applications.
• The presented method to stabilize the position of necking points during high-speed or low-speed drawing is of potential interest for the fiber community.
• The presented 2D WAXD fitting procedures based on different models are potentially useful to other researchers.
• The analysis of transversal and meridional scans of SAXS patterns is potentially useful to other researchers.
• The presented X-ray data is of great interest to the field of polymers and is useful for the further development of melt-spinning of PCL fibers.

## 1 Data Description

### 1.1 Properties of PCL polymer

#### 1.1.1 Rheological properties

**Fig. 1:**
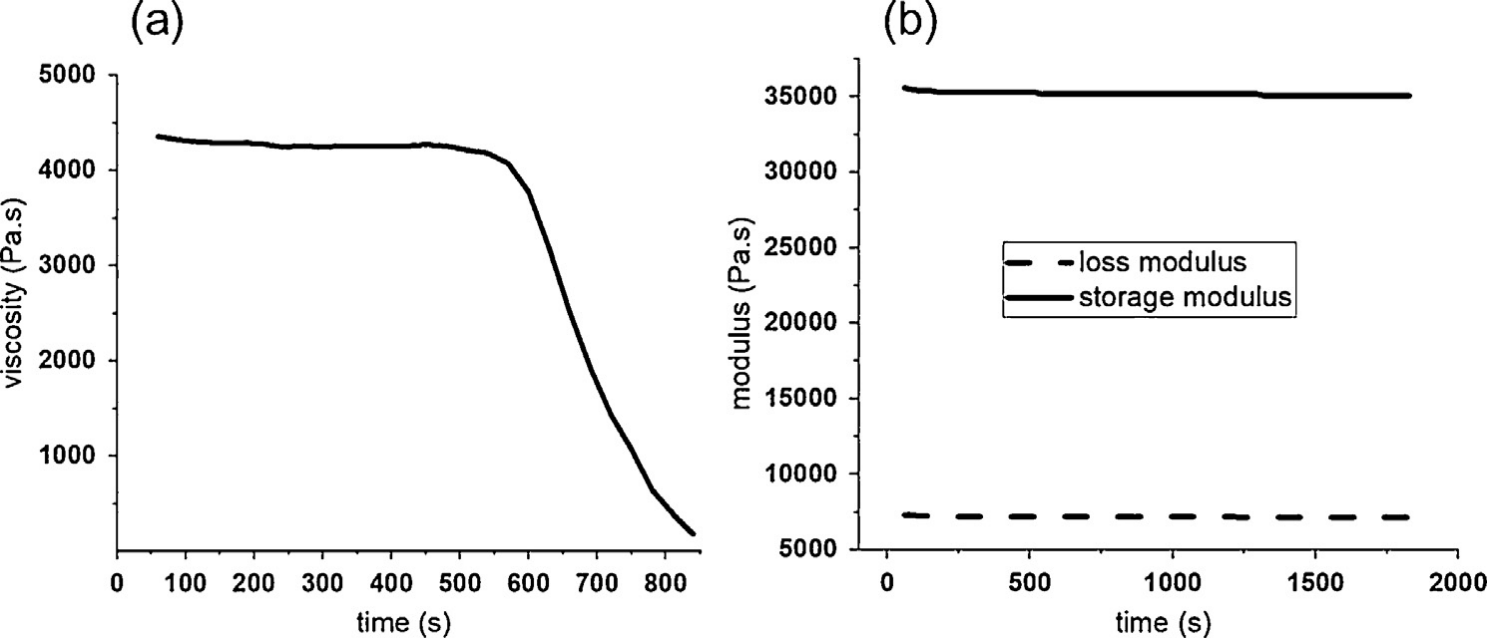
Rheological properties of PCL polymer: (a) measured viscosity as a function of time, (b) change in loss (*G*') and storage modulus (*G*'').

Time-dependent viscosity of the polymer was determined to be steady up to ten minutes, whereas the loss (*G*') and storage modulus (*G*'') were constant up to 30 min. Thus, no discernable degradation was observed at 80 °C (Fig. 1).

#### 1.1.2 Thermal properties of PCL

**Fig. 2:**
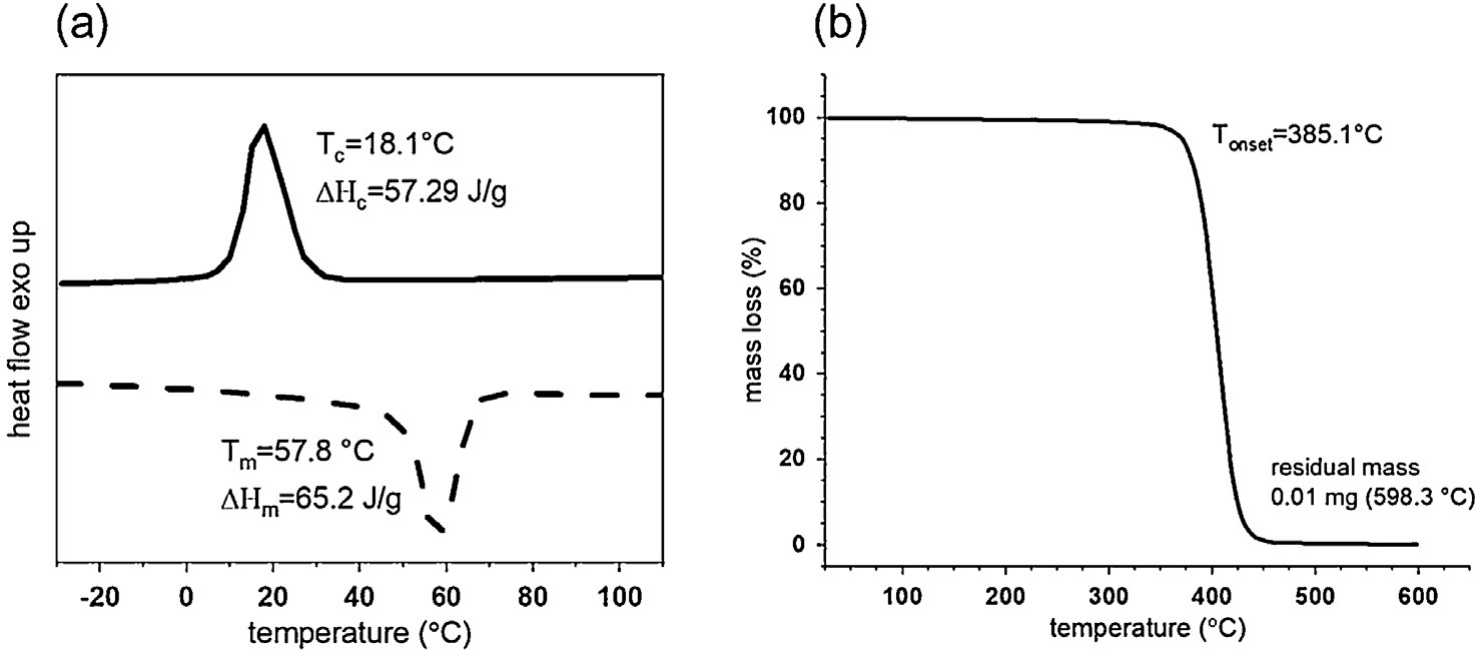
Thermal behaviour of PCL polymer: (a) cooling and second heating DSC curves, and (b) temperature-dependent mass loss of PCL, obtained from TGA.

Fig. 2a shows the 2nd heating and 1st cooling differential scanning calorimetry (DSC) curves and Fig. 2b shows the thermogravimetric analysis (TGA) curves of the PCL polymer (heating rates: 10 K/min). The melting temperature is 57.8 °C and the crystallization temperature is 18.1 °C. These values are in accordance with the data provided by the manufacturer. The melting enthalpy, ∆Hm= 65.2 J g^−1^ yields a crystallinity of 47% for the PCL polymer. Thermal stability of polymers during extrusion is crucial to enhance the processability and to prevent polymer degradation. TGA analysis reveals that PCL starts to degrade at 385.1 °C.

**Fig. 3:**
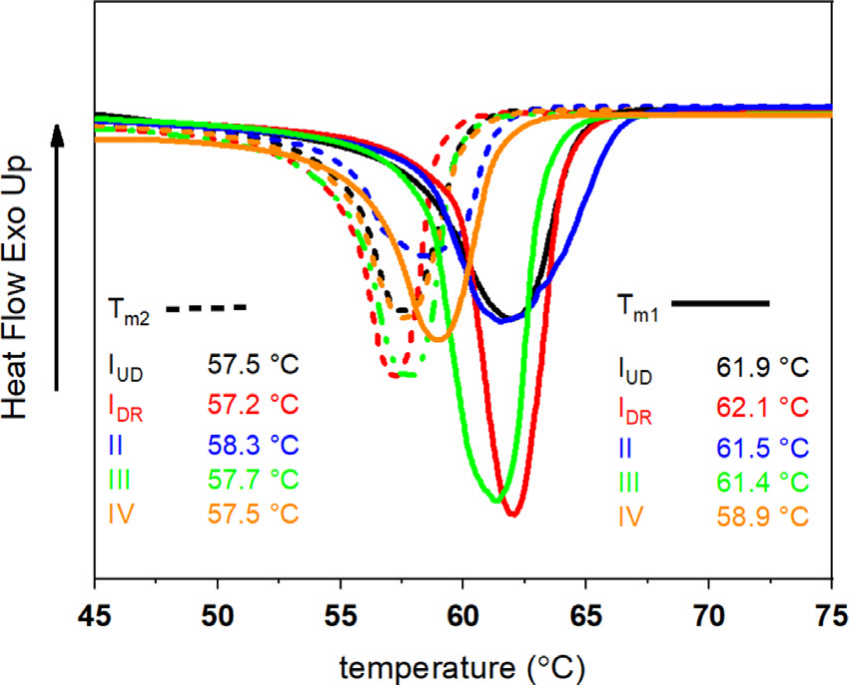
First (solid lines) and second (dashed lines) heating DSC curves of PCL filaments.

First and second heating DSC curves of PCL filaments are shown in Fig. 3. The first heating curves reflect the filament structure and the second heating curves reflect the structure of the polymer.

### 1.2 PCL filaments

#### 1.2.1 Drawing: necking stabilization on 2nd godet

**Fig. 4:**
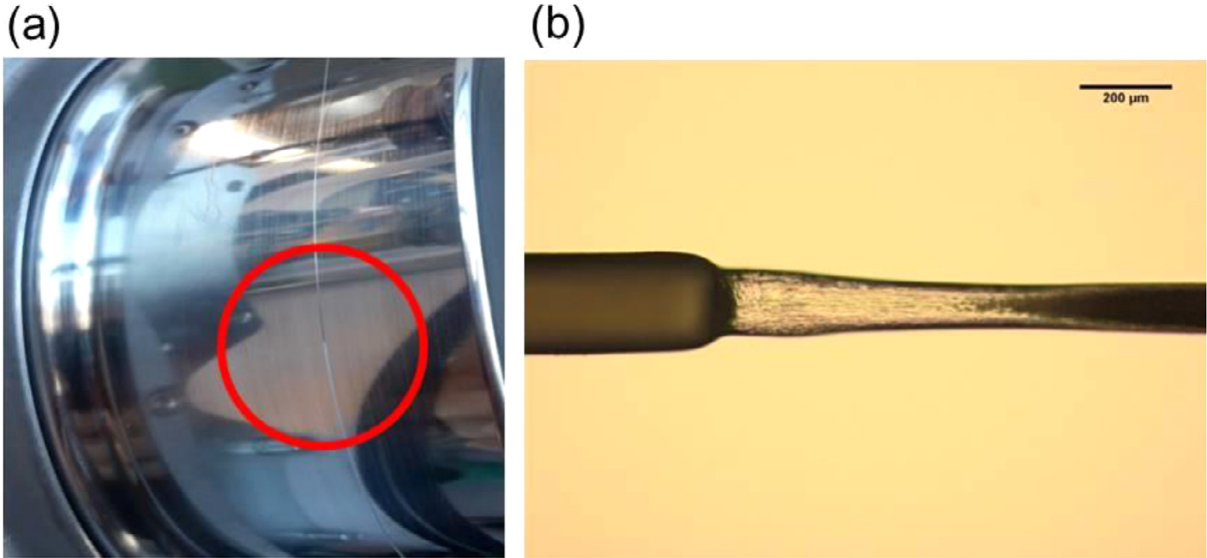
(a) Necking stabilization point on second godet. (b) Optical image of extension thinning behaviour of the monofilament (*I*_UD_) (magnification: 10×).

Fig. 4a shows the necking stabilization point on the second godet during drawing. The filament was guided over this godet without any windings and was running in reverse direction with respect to the rotation of the godet. The induced friction promoted the stress-induced necking and thus stabilized the position of the necking point. The optical image of the filament during drawing depicts its extension thinning behavior (Fig. 4b).

#### 1.2.2 Morphology

**Fig. 5:**
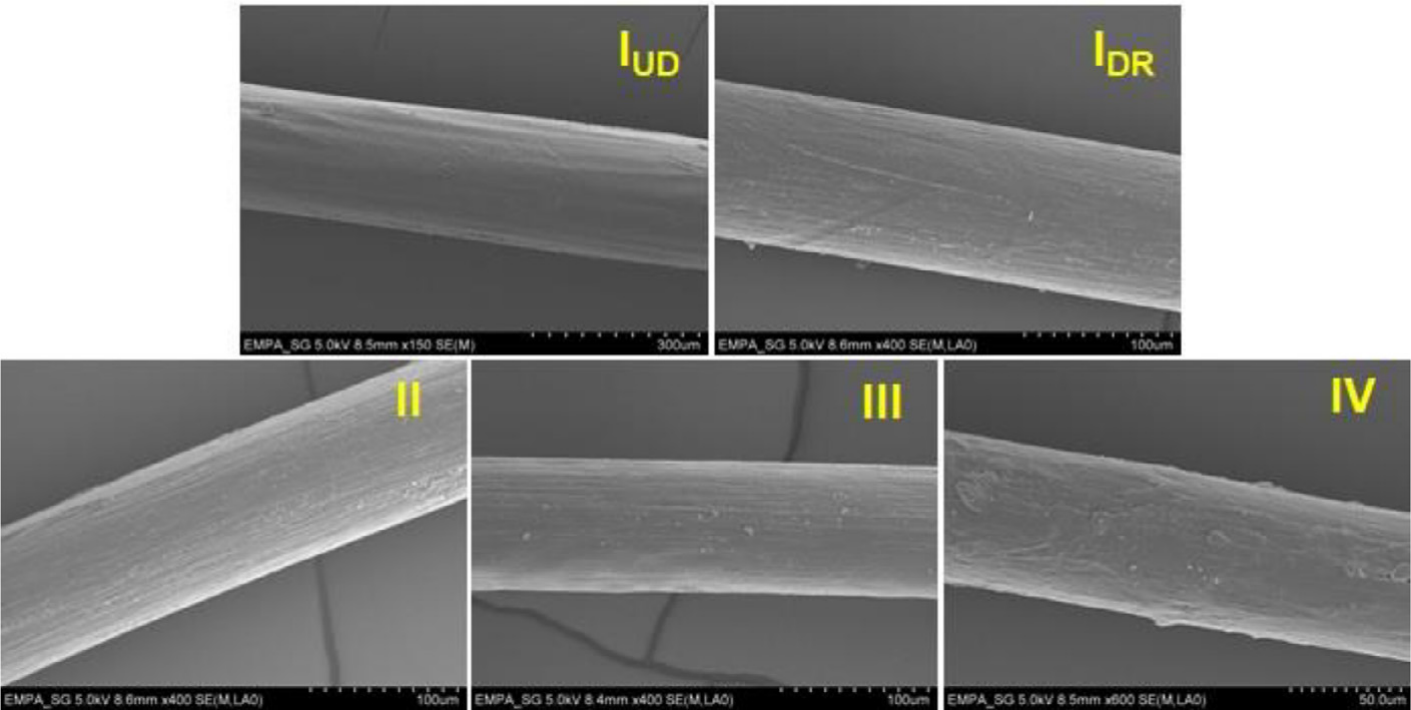
SEM images of PCL monofilament surfaces produced with different draw ratios (undrawn *I*_UD_ DR=1, offline drawn *I*_DR_ DR=7, and online drawn II, III and IV DR=6).

Fine PCL monofilaments <100 μm with smooth surfaces were successfully melt-spun. Fig. 5 shows the SEM images of all filaments. Online drawn filament IV has a rougher surface, which is most-likely due to a possible melt fracture/deformation because of the higher speed of the take-up godet (100 m/min).

#### 1.2.3 WAXD fitting results

Fitting errors for different models are summarized in Table 1.

**Table 1:** Fitting errors for different models. All fitting errors were multiplied by 10,000.

Fitting models	Description	Fiber	***χ***^2^	$\chi_{\mathrm{eq}}^{2}$	$\chi_{\left(110\right)}^{2}$	$\chi_{\left(200\right)}^{2}$
Best fit model 1	Unit cell: Chatani et al. [2] Amorphous Gaussian ring, mesophase *P*_nc_	*I*_DR_	3.67	1.47	0.87	1.33
		II	4.15	1.82	0.96	1.37
		III	3.41	0.92	0.85	1.64
		IV	5.08	1.92	1.09	2.06
Model 1a	Unit cell: Bittiger et al. [3] Amorphous Gaussian ring, mesophase *P*_nc_	*I*_DR_	6.82	3.34	1.39	2.08
Model 2	Unit cell: Chatani et al. [2] Amorphous Gaussian ring, two crystal sizes	*I*_DR_	6.33	2.05	1.72	2.57
Model 3	Unit cell: Chatani et al. [2] Amorphous Gaussian ring	*I*_DR_	5.95	1.89	1.75	2.31
		II	8.15	2.56	2.26	3.33
		III	7.36	2.67	1.81	2.87
		IV	9.10	2.73	2.37	4.01

**Fig. 6:**
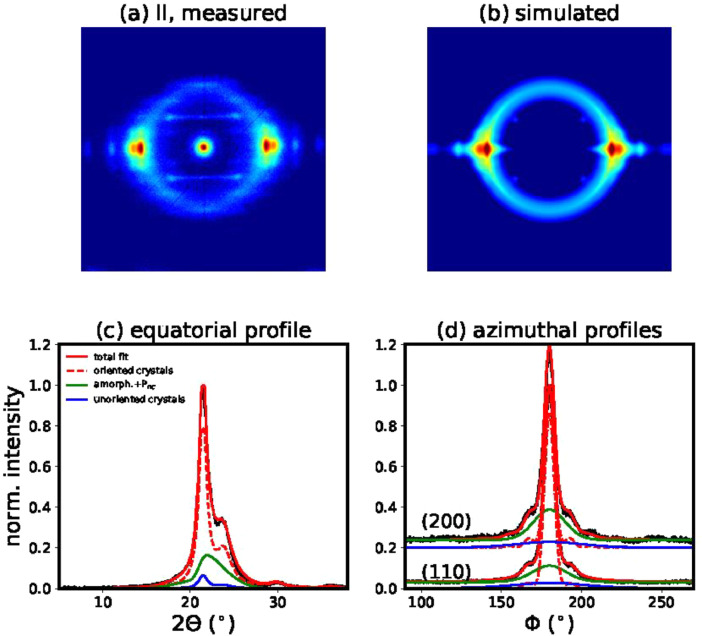
(a) Measured WAXD pattern of filament II. (b) Simulated WAXD pattern using the best fit parameters of model 1. (c) Normalized measured equatorial profile (black), best fit (red) and contributions from the oriented crystalline phase (dashed red) and amorphous plus mesophase (green) as well as from randomly oriented crystals (blue). (d) Normalized azimuthal profiles with the same colour coding. The azimuthal profile across the (200) reflection is offset for better visibility.

**Fig. 7:**
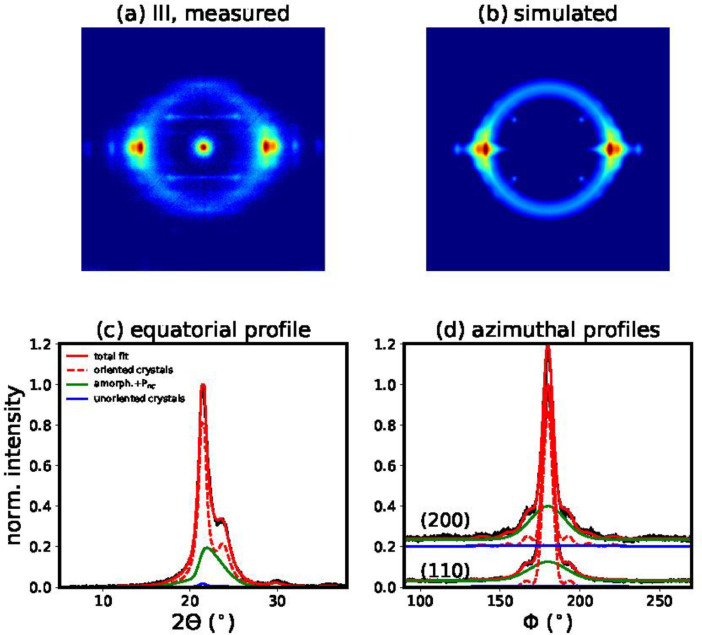
(a) Measured WAXD pattern of filament III. (b) Simulated WAXD pattern using the best fit parameters of model 1. (c) Fit of equatorial profile. (d) Fits of azimuthal profiles.

**Fig. 8:**
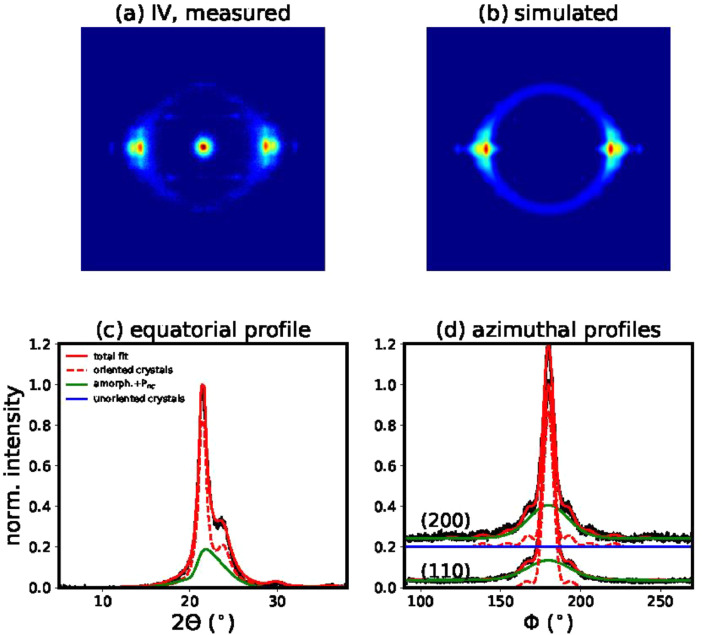
(a) Measured WAXD pattern of filament IV. (b) Simulated WAXD pattern using the best fit parameters of model 1. (c) Fit of equatorial profile (d) Fits of azimuthal profiles.

Best fit parameters that were obtained with model 1 are summarized in Table 2 for all drawn filaments. Corresponding figures of measured and best fit WAXD patterns, as well as fitted profiles are shown in Figs. 6–8 for drawn fibers II–IV.

**Table 2:** Best fit parameters for all drawn fibers.

Fibers	Crystalline phase						*P*_nc_ mesophase					Amorphous phase
	*p*	*p*_0_	Δ*x*_12_ (Å)	Δ*x*_3_ (Å)	*w*(200)	*w*(110)	*C*_norm_	*µ* (°)	*σ* (°)	Δ*x*_3_^Pnc^	*a*	*A*_norm_
*I*_DR_	379	0.104	0.61	1.02	0.027	0.017	2.27	20.9	2.6	3.70	5.0	0.95
II	352	0.311	0.10	2.00	0.035	0.017	2.07	21.0	2.6	4.30	5.0	1.08
III	365	0.107	0.75	1.41	0.030	0.017	2.52	21.0	2.3	2.76	5.0	1.08
IV	337	0.003	0.11	0.68	0.034	0.016	2.30	20.9	2.6	3.43	5.0	1.19

**Fig. 9:**
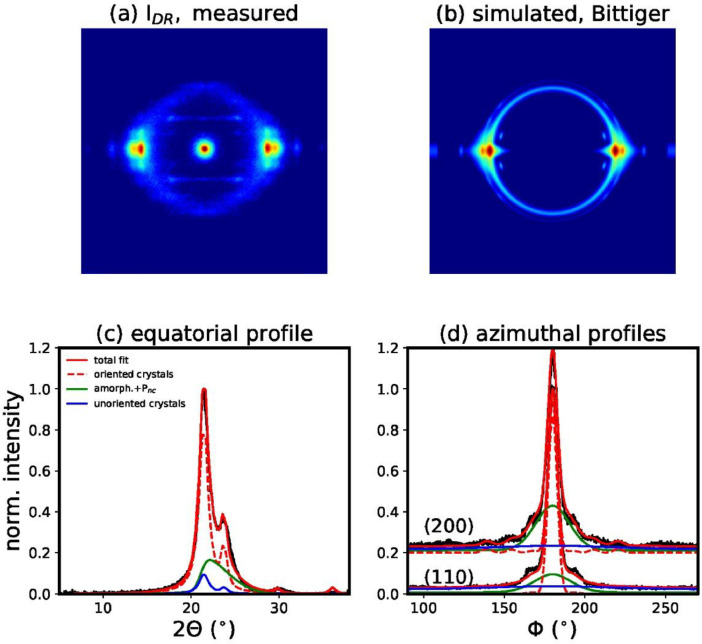
(a) Measured WAXD pattern of filament *I*_DR_. (b) Simulated WAXD pattern from fit parameters based on model 1a, with unit cell proposed by Bittiger et al. [3]. (c) Fit of equatorial profile. (d) Fits of azimuthal profiles.

**Fig. 10:**
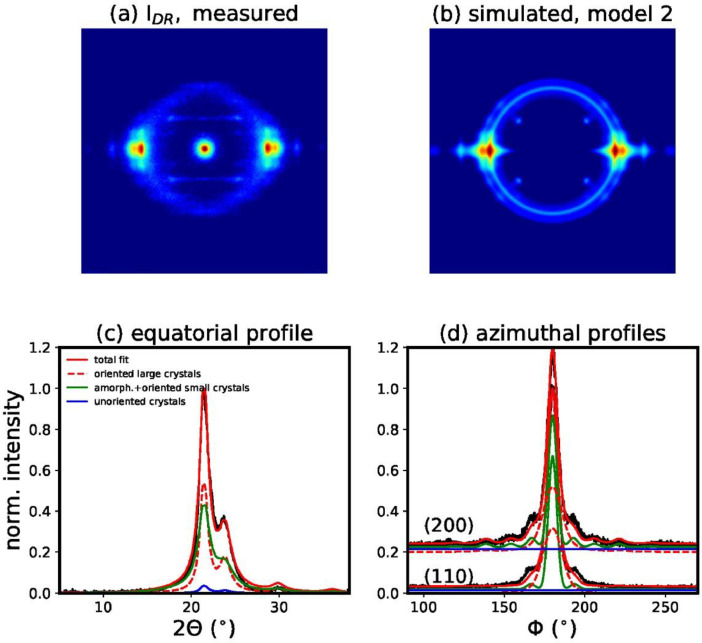
(a) Measured WAXD pattern of filament *I*_DR_. (b) Simulated WAXD pattern from fit parameters based on model 2, which assumes two different crystal sizes. (c) Fit of equatorial profile. (d) Fits of azimuthal profiles.

**Fig. 11:**
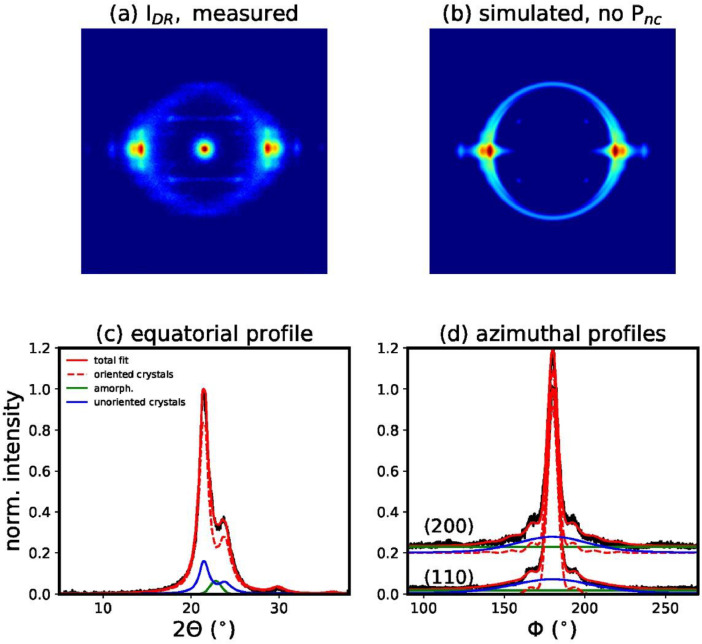
(a) Measured WAXD pattern of filament *I*_DR_. (b) Simulated WAXD pattern from fit parameters based on model 3, which neglects the mesophase. (c) Fit of equatorial profile. (d) Fits of azimuthal profiles.

We have also performed a fit of the measured WAXD pattern of filament I_DR_, with model 1a, which is based on the unit cell proposed by Bittiger et al. [3] (Fig. 9). The resulting fit error is significantly larger than for model 1. In order to exclude that the broader equatorial reflection underneath the main crystalline peaks arises from small crystals instead of a mesophase, we have also performed a fit, which is based on two crystal sizes, large, *l*, and small, *s* (model 2, Fig. 10). The model finds the following crystal sizes *D_l_*(200)=4.2 nm, *D_l_*(110)=6.2 nm and *D_s_*(200)=2.6 nm and *D_s_*(110)=3.8 nm. This model fits the equatorial profile reasonably well but the azimuthal profiles are not fitted well and the resulting fitting error is significantly higher than for model 1. Additionally, we have also performed a fit of the same pattern with model 3, which neglects the mesophase (Fig. 11). The fitting error for this model is also much larger (Table 1). Furthermore, the fit simulates a very narrow amorphous ring, which is unlikely to be real. Amorphous rings are typically quite broad due to the varying distances between chains. It becomes obvious that the fit is trying to artificially reproduce the mesophase, with the unoriented crystalline fraction, which is damped with a high damping factor away from the equator, and a narrow Gaussian ring. For simplicity, the theory uses the same DWF for the unoriented and oriented crystal fraction. In reality, it is however more probable that the unoriented part is not sensitive to the crystal imperfections along different directions.

#### 1.2.4 WAXD patterns of aged fibers

**Fig. 12:**
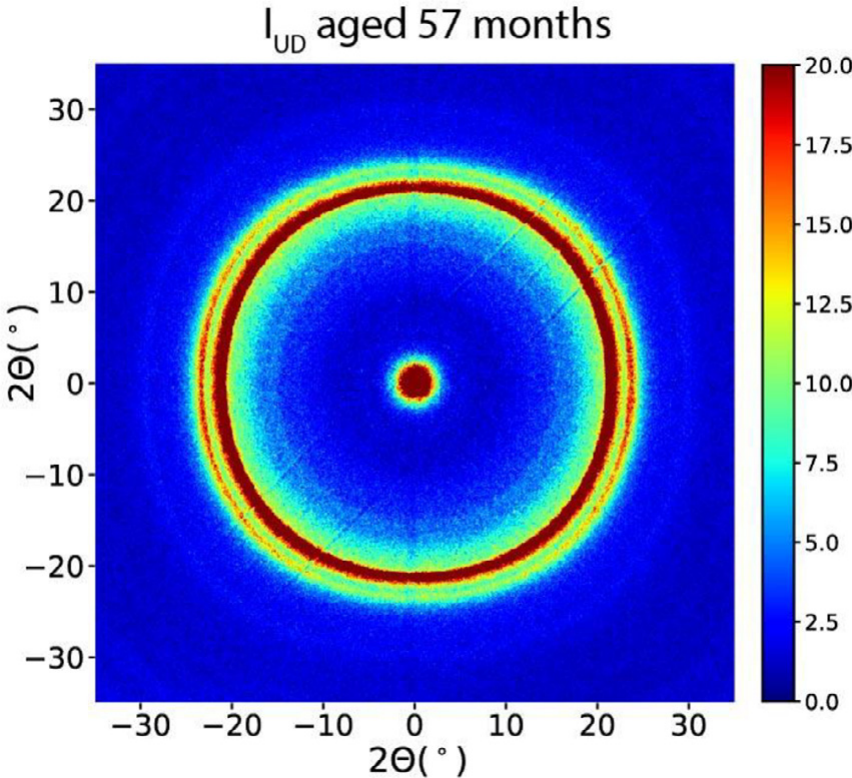
WAXD pattern of aged as-spun fiber.

**Fig. 13:**
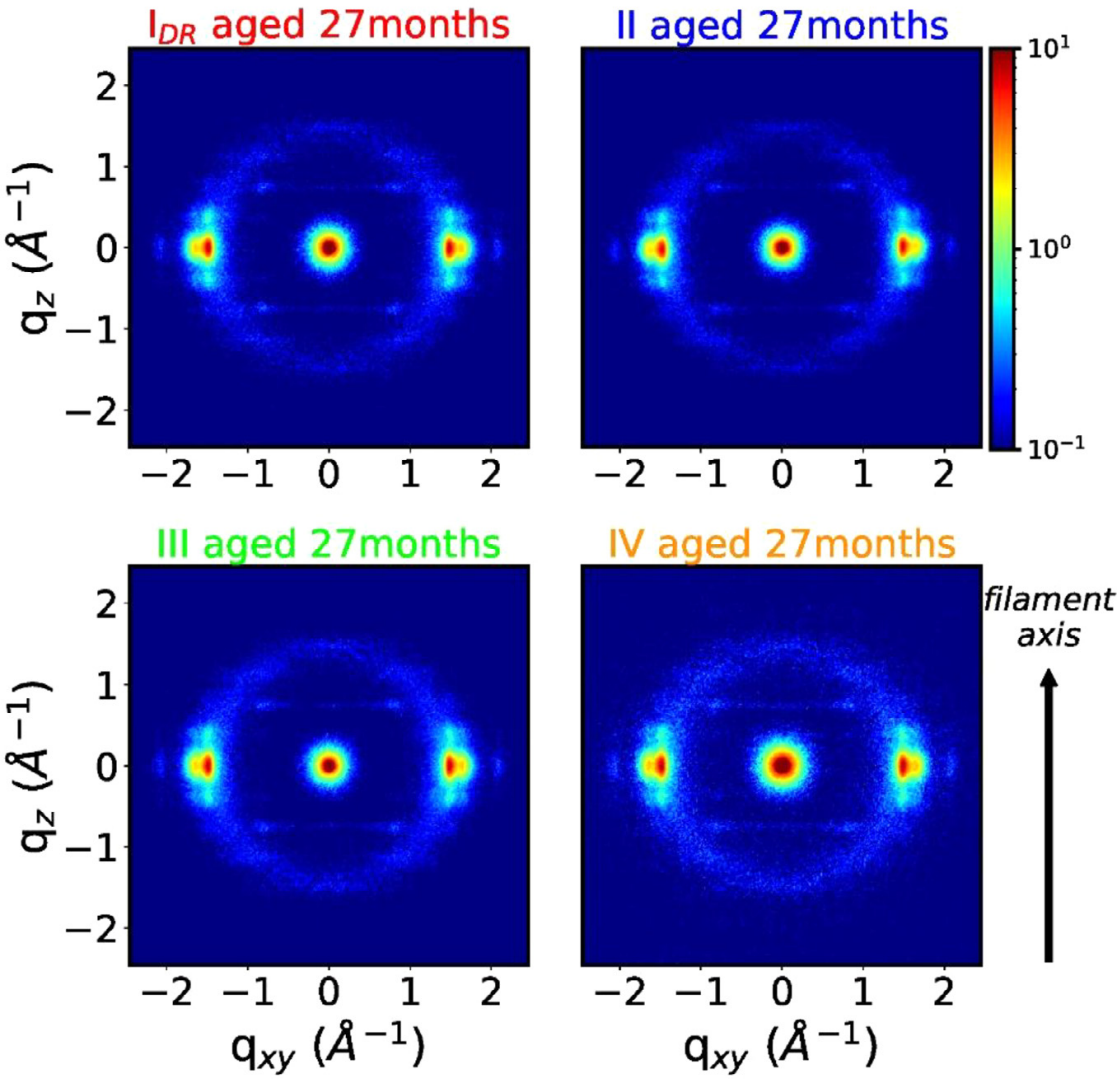
WAXD patterns of aged drawn fibers.

WAXD patterns of aged fibers are shown in Fig. 12 (as-spun) and Fig. 13 (drawn fibers).

#### 1.2.5 Direct crystallinity analysis from measured WAXD patterns

**Fig. 14:**
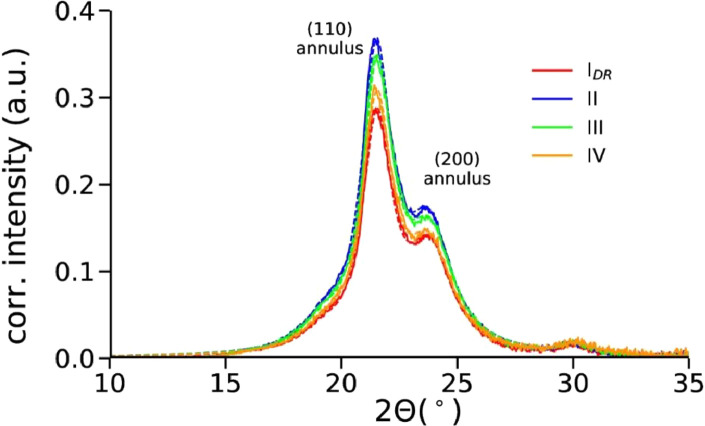
Radially integrated profiles with subtracted background. The measured data is shown as solid lines and the fits as dashed lines.

Radially integrated profiles were fit with Pearson VII functions using a shape factor *m*=1 and are shown in Fig. 14.

#### 1.2.6 SAXS analysis: meridional and transversal profiles

**Fig. 15:**
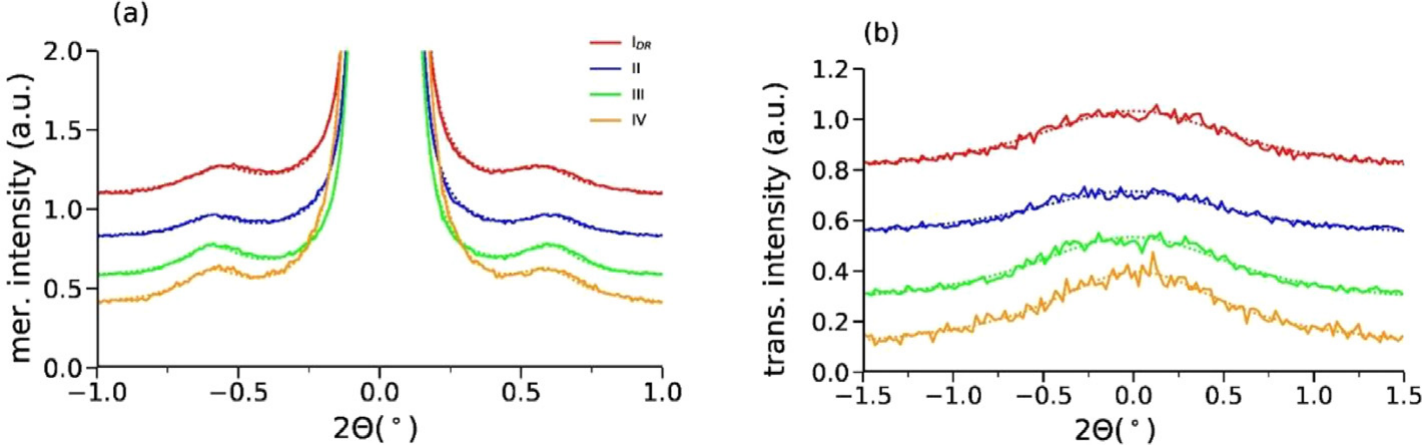
(a) Meridional and (b) transversal profiles. Measured data is shown as solid lines and fits are shown as dotted lines.

Meridional profiles and transversal profiles of the SAXS patterns are shown in Fig. 15.

## 2 Experimental design, materials, and methods

The materials and experimental methods for rheological as well as DSC/TGA measurements on PCL polymer and WAXD and SAXS on PCL fibers have been previously described in detail in the article by Selli et al. [1]. Part of the text below has been taken from the said article and is therefore put into quotes.

### 2.1 Materials

**'**Four PCL monofilaments were melt-spun from the PCL Capa^TM^ 6500 provided by Perstorp (UK) (Prochem AG, Zürich/Switzerland) in the form of pellets. The PCL (density of 1.1 g/cm^3^, nominal mean molecular weight of *M_w_*=50 kDa) had a melt flow index of 5.9–7.9 g/10 min (at 160 °C and a load of 2.16 kg) and a melting point of *T_m_=*58-60 °C. (..)'

### 2.2 Melt-spinning, online drawing and offline drawing of PCL filaments

**'**Melt-spinning of PCL monofilaments was carried out on a custom-made pilot-scale melt-spinning plant described elsewhere [4]. In total four filaments were melt-spun, an as-spun (undrawn) filament, *I*_UD_, as well as three online drawn filaments (II–IV). (..) DR=6 was the highest draw ratio that could be achieved for online drawn filaments without frequent fiber breakages. (..) The as-spun PCL filament (*I*_UD_) was subsequently drawn offline with the custom-made drawing and winding machine SSM RM3-T DIGICONE® preciflex^TM^ (Schärer Schweiter Mettler AG, Switzerland). For the resulting filament, *I*_DR_, a DR of 7 could be achieved.**'**

For more information about the setup and processing parameters, we refer the reader to the publication by Selli et al. [1].

### 2.3 Rheological measurements

Rheological properties of the PCL polymer were characterized with the Rheometer Physica MCR 301 (Anton Paar), using a plate-plate geometry. Time-dependent viscosity of the polymer was determined. Furthermore, the storage and loss moduli were measured as a function of time to investigate the degradation of the PCL polymer at 80 °C.

### 2.4 Differential scanning calorimetry (DSC) and thermogravimetric analysis (TGA)

'Thermal properties of PCL pellets and filaments were characterized using differential scanning calorimetry (DSC) and thermogravimetric analysis (TGA). Measurements with a DSC instrument (DSC 214 Polyma, Netzsch, Selb, Germany) were performed in nitrogen atmosphere (40 mL/min). The following heating and cooling cycles were applied: first heating from 0 °C to 120 °C, followed by a cooling step down to −50 °C and second heating back to 120 °C. The heating and cooling rates were set to 10 °C/min. TGA (TG 209 F1, Netzsch, Selb, Germany) was performed under nitrogen, increasing the temperature from 25 to 600 °C with a heating rate of 10 °C/min.'

### 2.5 Scanning electron microscope (SEM) imaging

The surface topography of fibers was analyzed using the scanning electron microscope (SEM) FE-SEM S-4800 (Hitachi High-Technologies Europe, Krefeld, Germany) with an acceleration voltage of 5.0 kV. All samples were coated with 8 nm gold prior to SEM measurements and longitudinal cross-sections of the fibers were examined.

### 2.6 WAXD and SAXS measurements

'WAXD and SAXS patterns were recorded on a Bruker Nanostar U diffractometer (Bruker AXS, Karlsruhe, Germany) with a Cu-Kα radiation λ = 1.5419 Å and a VÅNTEC-2000 MikroGap area detector. WAXD and SAXS measurements were performed several days after melt-spinning for the online drawn fibers and offline-drawn fiber. The as-spun fiber was measured 30 months after melt-spinning. Single filaments were used for all WAXD and SAXS measurements, which were performed in two separate experiments with distances of 19.1 cm and 110.1 cm, respectively, between the sample and the active detector area. The recorded WAXD/SAXS patterns were analyzed with the evaluation software DIFFRAC.EVA (version 4.2., Bruker AXS, Karlsruhe, Germany) and specifically developed python codes.' Raw (.gfrm) images from the Mendeley repository can be plotted with the open source Fabio python package.

### 2.7 WAXD analysis

#### 2.7.1 Intensity corrections

A background 2D pattern arising from air scattering was subtracted from the WAXD patterns. The intensities of the azimuthal and equatorial profiles were divided by the fineness of the filaments in order to account for the differences in the fiber diameter. For the measured equatorial profile, we subtracted a linear background, that arises most-likely from a weak equatorial streak. Before the fitting, the measured equatorial and azimuthal profiles were normalized by the strongest peak arising from (110) or (200) planes, respectively.

#### 2.7.2 Simulation and fitting of 2D WAXD patterns

The atomic form factors ***f_j_*** were calculated using the coefficients *a_i_, b_i_, c* from the international tables for crystallography [5] with the following equation:

(1) $$f_{j}\left(\left|s_{\mathrm{hkl}}\right|\right)=\sum_{i=1}^{4}a_{i}\exp\left(-b_{i}{\left(\frac{\left|s_{\mathrm{hkl}}\right|}{2}\right)}^{2}\right)+c$$

The scattered intensities were calculated as follows:

(2) $$I_{\mathrm{hkl}}\left(s_{\mathrm{hkl}}\right)=M_{\mathrm{hkl}}\;P\;{\left|F_{\mathrm{hkl}}\left(s_{\mathrm{hkl}}\right)\right|}^{2}$$

where *M_hkl_* are the multiplicity factors and *P* is the polarization factor, $[1+\text{co}s^{2}\left(2\theta \right)]/2$.

A Gaussian ring, ***I_ring_***, was added for the intensity contribution from the amorphous phase. Additionally, it was necessary to add a non-crystalline highly-oriented mesophase (*P_nc_*) intensity contribution, ***I_Pnc_***, in order to get good fits of the azimuthal and equatorial profiles. This phase was simulated with a strongly damped asymmetric Gaussian profile. For the damping, *DWF_Pnc_*, only an out-of-plane disorder parameter was taken into account and it was allowed to vary within certain bounds ($0\leq \Delta X_{3}^{Pnc}\leq 10\Delta X_{3}^{crystal}$). For simplicity, the width and the location of the mesophase were taken to be the same as the position and width of the amorphous phase.

(3) $$I_{\mathrm{sim}}=\bar{I\left(s,\varphi_{\mathrm{hkl}}\right)}\;F\left(\varphi ,\varphi_{\mathrm{hkl}}\right)\mathrm{DWF}\;+\;I_{\mathrm{ring}}+I_{\mathrm{Pnc}}$$

(4) $$\bar{I\left(s,\varphi_{\mathrm{hkl}}\right)}=\frac{1}{\left(4\pi w{\left|s_{\mathrm{hkl}}\right|}^{2}\right)}I_{\mathrm{hkl}}\left(s_{\mathrm{hkl}}\right)\frac{1}{1+{\left[\pi \left(s-s_{\mathrm{hkl}}\right)/w\right]}^{2}}$$

Finally, the overall intensity distribution of the 2D WAXD patterns, *I*_sim_, is described with the following functions:

(5) $$F\left(\varphi ,\varphi_{\mathrm{hkl}}\right)=p_{0}+\left(1-p_{0}\right)\left(\frac{p^{\prime }\cosh\left(p^{\prime }\cos\varphi \cos\varphi_{\mathrm{hkl}}\right)}{\sinh\left(p^{\prime }\right)}\right)I_{0}\left(p^{\prime }\sin\varphi \sin\varphi_{\mathrm{hkl}}\right)$$

(6) $$\mathrm{DWF}=\text{exp}\left[-\frac{4\pi^{2}}{3}\left(s_{12}^{2}\Delta X_{12}^{2}+s_{3}^{2}\Delta X_{3}^{2}\right)\right]$$

(7) $$I_{\mathrm{ring}}=A\exp\left[\frac{-{\left(2\Theta -\mu \right)}^{2}}{2\sigma^{2}}\right]\;P$$

(8) $$I_{\mathrm{Pnc}}=C\;\exp\left[\frac{-{\left(2\Theta -\mu \right)}^{2}}{2\sigma^{2}}\right]\;\left(1+\mathrm{erf}\left\{\frac{a\left(2\Theta -\mu \right)}{\sigma \sqrt{2}}\right\}\right)\mathrm{DW}F_{\mathrm{Pnc}}P$$

In order to exclude that the broader equatorial reflection underneath the main crystalline peaks arises from small crystals instead of a mesophase, we have also performed a fit which is based on two crystal sizes, 1/*w*_s_ and 1/*w*_l,_ where *s* stands for small and *l* for large crystals (model 2). In order to reduce the fitting parameters we introduced a proportionality factor, *w_s_*=k*w_l_. The oriented crystal fractions of these two different crystal sizes have different azimuthal widths *p'*_s,_ *p'*_l_. For simplicity of the fit, only an unoriented fraction, *p*_0_, of the larger crystals were taken into account and the same DWF was used for the oriented fractions (*p_s_* and *p_l_*). The following equations describe the fit with model 2:

(9) $$\bar{I_{2}\left(s,\varphi_{\mathrm{hkl}}\right)}=\frac{1}{\left(4\pi w{\left|s_{\mathrm{hkl}}\right|}^{2}\right)}I_{\mathrm{hkl}}\left(s_{\mathrm{hkl}}\right)\frac{1}{1+{\left[\pi \left(s-s_{\mathrm{hkl}}\right)/w_{s,l}\right]}^{2}}$$

(10) $$F_{2}\left(\varphi ,\varphi_{\mathrm{hkl}}\right)=p_{0}+\left(p_{s}+p_{l}\right)\left(\frac{p_{s,l}^{\prime }\cosh\left(p_{s,l}^{\prime }\cos\varphi \cos\varphi_{\mathrm{hkl}}\right)}{\sinh\left(p_{s,l}^{\prime }\right)}\right)I_{0}\left(p_{s,l}^{\prime }\sin\varphi \sin\varphi_{\mathrm{hkl}}\right)\mathrm{DWF}$$

(11) $$I_{\mathrm{sim}}=I_{\mathrm{ring}}+\bar{I_{2}\left(s,\varphi_{\mathrm{hkl}}\right)}\;F_{2}\left(\varphi ,\varphi_{\mathrm{hkl}}\right)$$

For model 2, we have used 10 fitting parameters for the crystalline part (*p_0_, p_s_, p_l_, p'_s_, p'_l_, w_l_, w_l_*(110), *k*,$\;\Delta X_{12}^{crystal}$ $\Delta X_{3}^{crystal}$and 3 for the Gaussian ring (*A, µ, σ*). In total, this amounts to 13 fitting parameters.

Model 3 excludes a mesophase and also small crystals and is based on the following equation:

(12) $$I_{\mathrm{sim}}=\bar{I\left(s,\varphi_{\mathrm{hkl}}\right)}\;F\left(\varphi ,\varphi_{\mathrm{hkl}}\right)\mathrm{DWF}\;+\;I_{\mathrm{ring}}$$

### 2.8 SAXS analysis

Long-spacing, *L*_3_, coherence lengths, *H*, as well as crystal sizes, *D*, were calculated by analyzing integrated meridional and transversal areas of the SAXS patterns. The lamellar long spacing, *L*_3_, is extracted from the meridional peak positions using the expression

(13) $$L_{3}=\frac{2\pi }{q_{\mathrm{LM}}}=\frac{\lambda }{\left(2\sin\theta_{\mathrm{LM}}\right)}$$

where $q_{LM}=\frac{4\pi }{\lambda }\sin\theta_{LM}$ is the scattering vector and *θ_LM_* is half the scattering angle at the lamellar reflection and λ is the X-ray wavelength. The coherence length *H* along the filament axis and lamellar stack sizes *D* perpendicular to the filament axis are calculated from the width of the lamellar reflections along the meridian and the width of the reflections in transversal scans using the Scherrer equation [6]

(14) $$\mathrm{size}=\frac{0.9\lambda }{\Delta \left(2\theta \right)\cos\theta }\approx \frac{0.9\lambda F}{\sqrt{\mathrm{FWH}M^{2}-b^{2}}}$$

where *size* stands for either the coherence length *H* or crystal size *D. F* is the filament-to-detector distance, *FWHM* is the full width at half-maximum of the reflection and *b* is the instrumental broadening, which is negligibly small (*b* ≈ 0). The equation makes use of small-angle approximations, cos *θ* ≈ 1.

## Declaration of Competing Interest

The authors declare that they have no known competing financial interests or personal relationships which have, or could be perceived to have, influenced the work reported in this article.
